# Pitfalls in the Modeling of Maltoside Detergents in Protein Structures

**DOI:** 10.1021/acscentsci.5c01861

**Published:** 2025-12-24

**Authors:** Sébastien Vidal, Louis F. L. Wilson

Structural biology aims to provide functional insight into biological macromolecules by deciphering the three-dimensional coordinates of their constituent atoms (albeit commonly in a low-energy state). Structural biologists deposit atomic coordinates—which may comprise polypeptides, polynucleotides, lipids, carbohydrates, and small molecule ligands—to the Protein Data Bank (PDB), wherein they become an invaluable and accessible resource to researchers from various fields. Many researchers from outside the structural biology field rely on the accuracy and integrity of this information, which has facilitated enormous progress in the biological sciences over the preceding decades.^1–3^

Increasingly, structures in the PDB are solved by single-particle cryo-electron microscopy (cryo-EM), a rapidly evolving technique that has gained huge popularity in recent times. Cryo-EM has enabled many important and groundbreaking discoveries that would have been previously impossible, albeit typically with lower resolution than the more traditional technique of X-ray crystallography. Regardless, researchers use the resultant density maps to build atomic models of the underlying macromolecules. However, whereas protein molecular models themselves are formulaic and supported by robust and easily accessible validation methods based on amino acid building blocks, it is more difficult to verify the structure of associated small molecule ligands and post-translational modifications, especially those based on saccharide moieties. Unfortunately, this can sometimes present the risk of errors in the interpretation of map features, particularly in the case of those that are low in resolution. Yet, this issue is seldom discussed in structural manuscripts, and atomic coordinates continue to be deposited without acknowledgment of the potential for modeling errors or bias.

Furthermore, in the case of carbohydrate-containing biomolecules, the fidelity and quality of coordinates have historically lagged behind those of polypeptides and polynucleotides, even in crystallographically determined coordinates.^4–6^ In many cases, conformationally and even stereochemically incorrect molecular structures have escaped the notice of editors and reviewers. Nevertheless, in recent times, glycoscientists have been working diligently to facilitate and automate glycan structural validation,^7^ and we hope that the quality of published glycan structures will soon begin to reflect this.

The determination of membrane protein structures has been greatly facilitated in recent times by, among other advances, the development of cryo-EM.^8^ Typically, membrane proteins are structurally characterized in the environment of a detergent micelle or nanodisc, which often populates the resultant map with many ambiguous nonprotein densities. These densities are usually assumed to correspond to structurally ordered (and sometimes functionally important) lipid and detergent molecules, and many investigators have attempted to model them atomically.

Owing to their mild nature, β-d-maltoside detergents are now the go-to choice for isolating membrane proteins for cryo-EM. In particular, *n*-dodecyl β-d-maltoside (DDM) and the dimaltoside detergents lauryl maltose neopentyl glycol (LMNG; Figure 1a) and glyco-diosgenin (GDN; Figure 1b) dominate the field.^9,10^ Given their widespread use, we had assumed that their structures would be well-known and thus uniformly presented in the academic literature. Surprisingly, however, we have discovered that, of all PDB structures attempting to model full-length detergent molecules, half of those claiming to model LMNG and an astonishing 27 out of 28 claiming to model GDN contain the wrong molecule (Table 1). In almost all these cases of error—which have mainly arisen during the last five years—an unnatural l-maltoside/l-glucosyl-l-mannoside ‘near-enantiomer’ has been modeled instead of LMNG (Figure 2), while an α-maltoside stereoisomer has been modeled instead of GDN (Figure 3).

**Figure 1:**
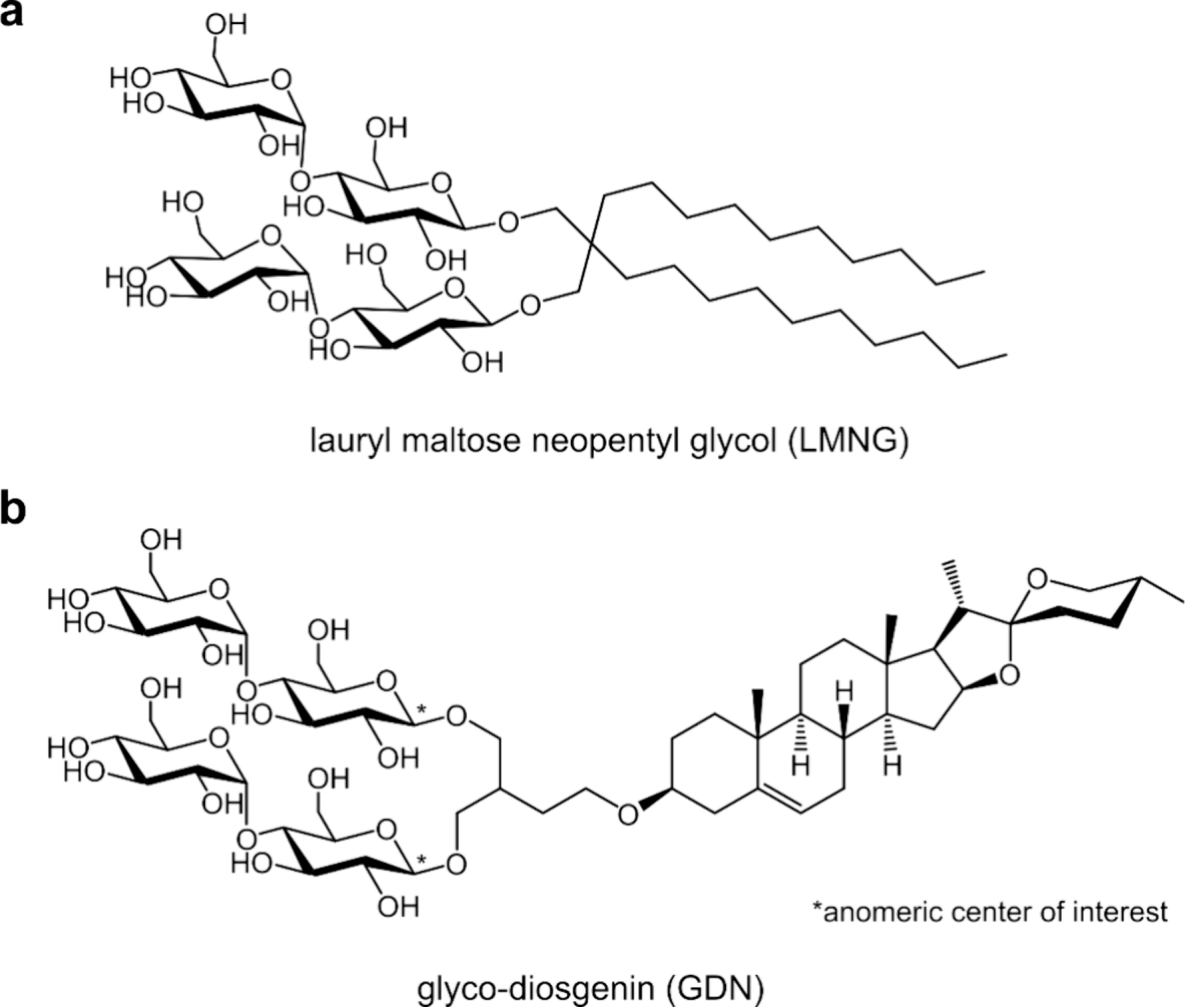
Modern β-maltoside detergents. (a) Structural formula of lauryl maltose neopentyl glycol (LMNG). (b) Structural formula of glyco-diosgenin (GDN).

**Table 1:** List of Full-Length (or Close to Full-Length) Models of LMNG and GDN in the Protein Data Bank

molecule		PDB ligand ID	PDB structures including this molecule	relevant figures
Molecules Used to Represent LMNG				
LMNG (correct structure)		AV0	6IIA, 6VP0, 7MDF, 7NVH, 7ONJ, 7OOA, 7RPH, 7RPI, 7RPK, 8HKP, 8J74, 8JEW, 8JEZ, 8JF1, 8JZN, 8SGW, 8SH3, 8SHC, 8SIE, 8U4 V, 8U5B, 8UUK, 8W1 V, 8XM6, 8XMA	
lauryl ‘l-maltose/l-glucosyl-lmannose’ neopentyl glycol				
	10.1038/nature11683	LMN	4B4A	2, 4, S2, S5
	10.1126/sciadv.aba4996	LMN	6L3T, 6L3U, 6L3V	S2, S10
	10.1126/sciadv.aax9484	LMN	6RFQ, 6RFR	3, S2
	10.1038/s41467-019-11977-1	LMN	6S8H, 6S8N	S8
	10.1038/s41594-019-0357-0	LMN	6SP2	
	10.1016/j.celrep.2020.107837	LMN	6WQZ, 6WR4	S4
	10.1038/s41467-020-19778-7	LMN	6Y79	
	10.1093/plcell/koab092	LMN	7AQQ, 7AR7, 7AR8, 7ARB	S9
	10.1038/s41594-020-00518-w	LMN	7D0I	ED4
	10.1126/sciadv.abj3221	LMN	7O6Y, 7O71	
	10.1126/sciadv.abn8063	LMN	7SK8	
	10.1038/s41467-022-32831-x	LMN	7Z0S	
	10.7554/elife.90174.3	LMN	7ZKA, 7ZKQ	
	10.1126/sciadv.adc9952	LMN	7ZMB, 7ZME	2
Molecules Used to Represent GDN				
GDN (correct structure)		YJ0	8T1B	
α-GDN (anomer)				
	10.1073/pnas.2011560117	Q7G	7K65	2, S4
	10.1038/s41467-022-28984-4	Q7G	7TJ9	
	10.1038/s41467-022-33588-z	Q7G	8AP6, 8AP7, 8APA, 8APB, 8APC, 8APD, 8APE, 8APF, 8APG, 8APH, 8APJ, 8APK	S6
	10.1038/s41477-022-01308-6	Q7G	8BEF, 8BEL, 8BPX	4
	10.1038/s41467-023-41747-z	Q7G	8C80, 8C81, 8C82	3
	10.1016/j.bbamcr.2023.119543	Q7G	8IJL	2
	10.1016/j.str.2024.05.005	Q7G	8OUO	
	10.1073/pnas.2315575121	Q7G	8QEC	2, S7
	10.1016/j.celrep.2024.114627	Q7G	8QOF, 8QOG	3, S4
	10.3390/ijms252212397	Q7G	8ZYJ	1–4
d-Glc-β-1,4-l-All-β-(dMan-β-1,4-l-Glc-β-)-isoamyl triol-isodiosgenin				
	10.1038/s41467-021-23248-z	J4U	7DUW	S3

**Figure 2:**
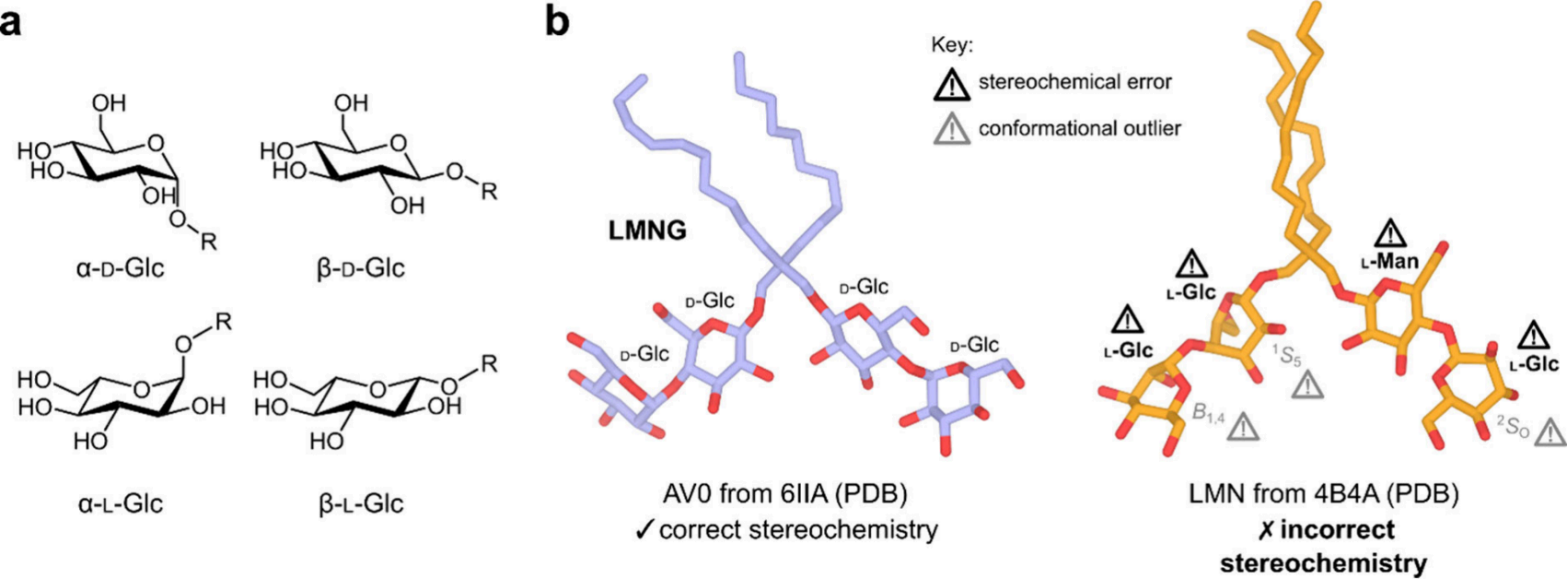
Anomers and enantiomers of glucose and some examples of correct and incorrect LMNG models in the PDB. (a) Structural formulas for glucopyranose showing both enantiomers (l- and d-) and anomers (α and β). (b) An example of a correctly modeled LMNG molecule from Protein Data Bank (PDB) entry 6IIA (left) compared with an incorrect l-glucosyl near-enantiomer from PDB entry 4B4A (right). Glc = glucose; Man = mannose. Outlier conformations for each pyranose ring are designated according to Cremer–Pople notation.

**Figure 3:**
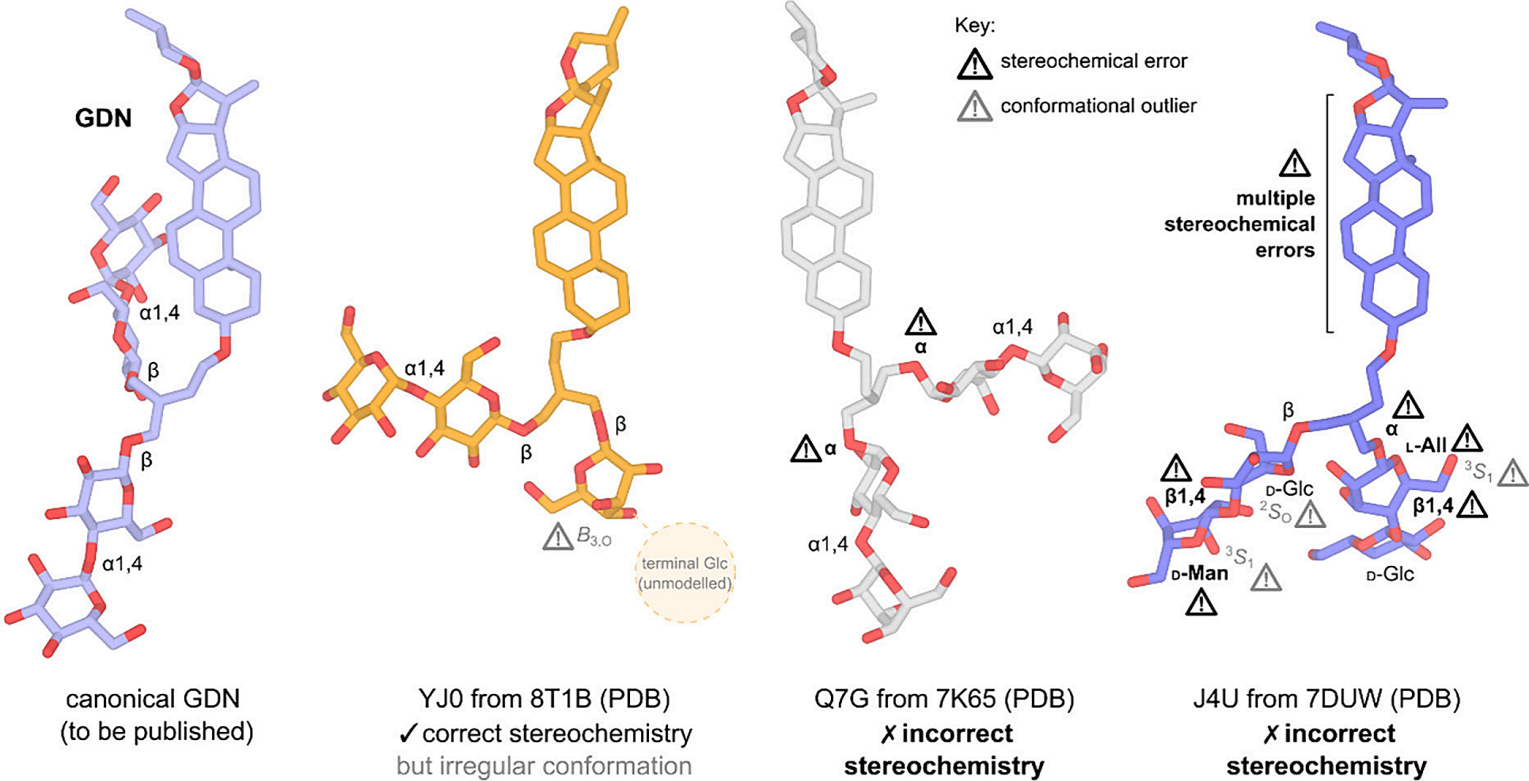
Some examples of correct and incorrect GDN models in the PDB. Examples: a GDN model without any stereochemical or conformational errors (unpublished, left), a model with correct stereochemistry but unlikely conformation from PDB entry 8T1B (second from left), a model with incorrect α-maltosyl anomers from PDB entry 7K65 (third from left), and a model with gross stereochemical errors (including incorrect epimers and anomers) from PDB entry 7DUW (right). Glc = glucose; Man = mannose; All = allose. Outlier conformations for each pyranose ring are designated according to Cremer–Pople notation.

The distinction between such isomers is functionally important. For example, the α-maltosyl variety of DDM (‘α-DDM’) displays substantial differences in micelle shape^11^ and solubilization properties^12–14^ compared with the more commonly used β-DDM. Furthermore, some of the publications we reviewed highlighted detergent binding as a key point of interest and purported to illustrate the binding site in molecular detail. Even more troubling is the fact that in many cases, investigators imposed energetically infeasible conformations (i.e., puckering of the pyranose rings) in order to force erroneous molecules into the experimental map. This raises further questions as to whether the densities themselves have been interpreted correctly in the first place.

It is likely that these particular issues have arisen through error propagation (specifically by reuse of the PDB ligands with entry IDs of LMN and Q7G, respectively). We also note that the structural formula for GDN was corrected after its initial publication,^15^ which could give rise to further confusion. We urge previous authors to review the chemical structures of LMNG and GDN and correct their coordinates and figures accordingly, not only for the reasons above but also so that these errors do not continue to spread through the field.

We would like to use the above examples to raise awareness of carbohydrate chemistry and to help authors avoid more serious scientific errors in the future. Minor structural variations between different carbohydrate molecules result in vast differences in their biological properties; be they immunological (e.g., cell surface receptors, blood groups, etc.) or rheological (e.g., cellulose vs starch). Structural biologists, in particular, should exercise caution when building and refining carbohydrate ligands, especially in the absence of appropriate restraints. We recommend the use of the Privateer web app (https://privateer.york.ac.uk/, York Structural Biology Laboratory) and/or the Cremer–Pople Parameter Calculator (https://enzyme13.bt.a.u-tokyo.ac.jp/CP/, Shinya Fushinobu) to help validate glycan conformation.

Here, we have focused on a single class of ligand, and we hope that our findings are not representative of the PDB at large. While the biophysical techniques underlying structural biology are not in question, we call on authors, peer-reviewers, and editors to exercise their due diligence in adequately verifying and reporting ligand structures as well as qualifying their experimental support. It is only by doing so that we can fully safeguard the integrity of structural data, especially when it comes to carbohydrates and other ligands.
